# Boosting hypochlorite’s disinfection power through pH modulation

**DOI:** 10.1186/s12866-025-03831-w

**Published:** 2025-02-28

**Authors:** Timir Baran Sil, Dmitry Malyshev, Marina Aspholm, Magnus Andersson

**Affiliations:** 1 Department of Physics, Umeå University, Umeå, 90187 Sweden; 2 Department of Paraclinical Sciences, Norwegian University of Life Sciences, Ås, 1432 Norway; 3 Umeå Centre for Microbial Research (UCMR), Umeå University, Umeå, 90187 Sweden

**Keywords:** Decontamination, Spores, Raman, NaOCl, HOCl, *Bacillus*

## Abstract

**Purpose:**

Hypochlorite-based formulations are widely used for surface disinfection. However, the efficacy of hypochlorite against spore-forming bacteria varies significantly in the literature. Although neutral or low pH hypochlorite solutions are effective sporicides due to the formation of hypochlorous acid (HOCl), their optimal conditions and the specific role of pH in disinfection remain unclear. These conditions also increase the solution’s corrosiveness and compromise its shelf life. Therefore, further research is needed to identify the pH conditions that balance solution stability and effective hypochlorite-based spore disinfection.

**Results:**

This study investigates the impact of neutral to alkaline pH on the sporicidal efficiency of hypochlorite against a pathogenic *Bacillus cereus* strain. We apply a 5,000 ppm hypochlorite formulation for 10-min across a pH range of 7.0-12.0, simulating common surface decontamination practices. Our results demonstrate that hypochlorite is largely ineffective at pH levels above 11.0, showing less than 1-log reduction in spore viability. However, there is a significant increase in sporicidal efficiency between pH 11.0 and 9.5, with a 4-log reduction in viability. This pH level corresponds to 2 - 55 ppm of the HOCl ionic form of hypochlorite. Further reduction in pH slightly improves the disinfection efficacy. However, the shelf life of hypochlorite solution decreases exponentially below pH 8.5. To explore the pH-dependent efficacy of hypochlorite, Raman spectroscopy and fluorescence imaging were used to investigate the biochemical mechanisms of spore decontamination. Results showed that lower pH enhances spore permeability and promotes calcium dipicolinic acid (CaDPA) release from the core.

**Conclusion:**

Our results highlight the complex relationship between pH, sporicidal efficacy of hypochlorite, and its shelf life. While lower pH enhances the sporicidal efficiency, it compromises the solution’s shelf life. A pH of 9.5 offers a balance, significantly improving shelf life compared to previously suggested pH ranges 7.0-8.0 while maintaining effective spore inactivation. Our findings challenge the common practice of diluting sodium hypochlorite with water to a 5,000 ppm solution, as this highly alkaline solution (pH of 11.9), is insufficient for eliminating *B. cereus* spores, even after a 10-min exposure. These findings are critical for improving disinfection practices, highlighting the importance of optimizing sodium hypochlorite effectiveness through pH adjustments before application.

**Supplementary Information:**

The online version contains supplementary material available at 10.1186/s12866-025-03831-w.

## Background

Bacterial spore contamination is a major challenge in the food industry and healthcare sectors [1]. Contamination by species within the *Bacillus cereus group* is a frequent cause of foodborne disease outbreaks and substantial economic losses due to product spoilage [2]. Moreover, spore contamination in hospitals can result in serious illnesses, such as *Clostridiodes difficile* infection. These problems are particularly problematic in developing countries, with issues such as surgical equipment contamination, which can cause life-threatening infections [3]. To safeguard public health and food safety, effective disinfection of spore-contaminated surfaces is crucial. Unfortunately, bacterial spores are highly resistant to many common disinfection methods, including high and low pH, heat, ethanol, and ammonium-based agents [4, 5].

Sodium hypochlorite (NaOCl), the active ingredient in common household bleach and hospital disinfectants, is one of the most widely used disinfectants worldwide [6]. As a chlorine-releasing agent (CRA), it effectively degrades biological matter, including bacterial spores, through a process known as chloramination of amino acids [7–9]. The inactivation of spores by sodium hypochlorite is assumed to occur through the penetration of hypochlorous acid (HOCl) into the spore. Once inside, HOCl directly attacks the inner membrane, DNA, and other cellular components [10, 11]. Thus, the chemical inactivates spores more rapidly than it degrades their physical structure. For spores of *Bacillus* species, the sporicidal activity has been reported to be most effective at “physiological” or “neutral” pH values (approximately 7–8). At higher pH levels (8–9), the sporicidal efficacy rapidly decreases [12]. While using hypochlorite within the lower pH range enhances its sporicidal activity, it also shortens its shelf life [13]. The reason for the shortened lifetime is that at lower pH levels, HOCl becomes less stable and readily decomposes into sodium chlorate, eventually breaking down further into chlorine and water [14, 15].

The decomposition rate is further controlled by the temperature, concentration, and pH of the solution [14]. At high pH (pH >9), the major decomposition reaction product of hypochlorite is the chlorate ion, while oxygen is formed as a side product as described in the following reactions,

1 $$\begin{aligned} {3\,\text {NaOCl} \longrightarrow \text {NaClO}_{3} + 2\,\text {NaCl}} \end{aligned}$$

2 $$\begin{aligned} {2\,\text {NaOCl} \longrightarrow \text {O}_{2} + 2\,\text {NaCl}} \end{aligned}$$

When the pH is in the range of 5 to 9, HOCl begins to form and interacts with the hypochlorite ion, leading to its decomposition according to the following reaction,

3 $$\begin{aligned} {2\,\text {HOCl} + \text {NaOCl} \longrightarrow \text {NaClO}_{3} + 2\,\text {H}^{+} + 2\,{\text {Cl}}^{-}} \end{aligned}$$

Further reduction in the pH of the solution (pH <5) leads to the decomposition of hypochlorite to chlorine gas and water.

4 $$\begin{aligned} {\text {ClO}^{-} + 2\,\text {H}^{+} + \text {Cl}^{-} \longrightarrow \text {Cl}_{2} + \text {H}_{2}\text {O}} \end{aligned}$$

Hence, to maintain a longer shelf life, commercial hypochlorite solutions are typically supplied in an alkaline form with a pH > 12.

The complex chemistry of hypochlorite decomposition at different pH values and concentrations over time greatly affects the effectiveness of the solution in killing spores. As such, the reported sporicidal efficacy of hypochlorite solutions varies widely and direct comparisons of results from different studies are challenging due to variations in experimental methodologies. In Table 1, we summarize the literature on the effectiveness of hypochlorite against *Bacillus* spp. spores at different concentrations and under different conditions. For example, Death et al. reported that a 10-min exposure to 100 ppm hypochlorite resulted in a >5-log reduction in viable spores at pH 8 and below. However, at pH levels of 9 or higher, the reduction was <1-log [12]. Bloomfield et al. reported a 3.7-log reduction in viable spores after a 5-min exposure to 200 ppm hypochlorite at pH 7 [16]. Sabli et al. observed that 10,000 ppm hypochlorite, without pH adjustment, did not affect *B. subtilis* spores even after 30-min of incubation [17]. Young et al. reported that at pH 11, 2,500 ppm hypochlorite was largely ineffective (<1-log) in killing spores unless very long incubation times were used. In contrast, at pH 7, a concentration as low as 50 ppm achieved a 5-log reduction in viable spores within 10-min [10]. Verguet and colleagues used 96,000 ppm hypochlorite on *B. anthracis* (earlier studies used *B. subtilis*) in simulated dirty conditions (0.3% bovine serum albumin) without pH adjustments and obtained a 4.7-log reduction in the number of viable spores within 8-min [18]. Thus, the hypochlorite concentrations tested across studies vary greatly, ranging from as low as 50 ppm to as high as 96,000 ppm, compared to the typical concentration of approximately 50,000 ppm found in undiluted domestic bleach. This variability underscores the complex interplay between pH and the sporicidal activity of hypochlorite. In contrast to the trend observed for *Bacillus* spores, *C. difficile* spores were found to be more resistant to hypochlorite at neutral pH, with higher efficacy observed under alkaline pH [19]. Overall, these reported results show great variability and many of them do not meet the existing industrial standards like EN 17126, which requires a 4-log reduction in no more than 15 min [20].

**Table 1:** Effectiveness of hypochlorite in reducing *Bacillus* spore counts under various concentrations, pH levels, and exposure times

PPM	pH	Time (min)	Log Reduction	Notes	Ref.
50	7	10	5	*B. subtilis*	[10]
100	5.2 - 9.9	10	>5 (at pH$\le$8), <1 (at pH$\ge$9)	*B. subtilis*. Most data at pH 7.0 - 8.4	[12]
140	Stock	10	2	*B. subtilis*	[21]
200	7	5	3.7	*B. subtilis*	[16]
2,500	11	10	<1	*B. subtilis*	[10]
10,000	Not reported	30	0	*B. subtilis*	[17]
1,000	7	10	4.2	*B. anthracis*	[22]
5,500	Not reported	20	4	*B. anthracis*	[23]
96,000	Stock	8	4.7	*B. anthracis*	[18]
243	Stock	15	>3	*B. cereus*. Sporicide added to wash rinse cycle	[24]
25,000	7	10	4–5	*B. subtilis* dried on nonporous materials	[25]
3–4.5	7	240	<1 (10 min), $\approx$6 (240 min)	*B. subtilis*. Uses a combined unit for exposure, mg min L^−1^ of chlorine (concentration $\times$ time)	[26]
2,000	10.47	10	0	*Alicyclobacillus spp.*	[27]

“Stock” refers to commercial hypochlorite formulations without pH values reported

The inconsistencies in the reported sporicidal efficacy of hypochlorite highlight a significant knowledge gap in understanding the precise mechanisms behind spore inactivation. Additionally, there is a limited understanding of how pH influences hypochlorite’s sporicidal activity, including the optimal pH that maximizes spore killing while maintaining solution stability.

In this study, we investigate the relationship between pH, sporicidal efficacy, and the shelf life of sodium hypochlorite. Our findings reveal a critical transition, with a 4-log difference in spore reduction between pH 9.5 and 11.0, challenging previous reports that identified neutral pH as the decisive range. Sporicidal activity begins at <2 ppm HOCl and reaches a 4.1-log reduction in 10-min at 55 ppm. Lowering the pH further, from 9.2 to 7.4, increased spore inactivation from 4.1-log to 5.0-log but substantially compromised the hypochlorite solution’s shelf life. Altogether, these results contribute to safer and more efficient disinfection protocols in food production and healthcare environments where spore contamination presents persistent challenges.

## Methods

### Materials

We used a commercially available sodium hypochlorite solution (Klorin Original, 200000043775, Colgate-Palmolive AB) for the experiments. The hypochlorite solution contains sodium hydroxide as a pH-stabilizing agent, but it does not contain any other chemicals that could interfere with its sporicidal activity. We adjusted the pH of the hypochlorite solution using hydrochloric acid (HCl) (20252.290, VWR). The quenching of hypochlorite activity was performed using sodium thiosulfate (217263, Sigma-Aldrich).

### Strains, growth and purification

We obtained the *B. cereus* NVH 0075/95 strain, also referred in the literature as *B. paranthracis*, from the Norwegian University of Life Sciences. To prepare the spore stock, we grew the bacteria on tryptic soy agar (TSA, Bacto^TM^, BD) at 37°C for one week to induce sporulation [28].

The spores were collected and purified as described previously [29, 30]. We harvested spores from plates, suspended them in autoclaved deionized water, and washed them by centrifugation and resuspension (4,500 g, 5 min, 20°C) in deionized water. The spore stock was stored in deionized water at 4°C overnight. The next day, the suspension was heat shocked at 85°C to kill the remaining vegetative cells. The washing step was repeated three times, followed by layering the suspension onto a 20% Histodenz (D2158, Sigma-Aldrich) solution and centrifuging at 3,400 g for 20 min at 20 °C. The suspension was washed three more times to remove the Histodenz. The final pellet was resuspended in deionized water and stored at 4°C. The purity of the spore suspension was verified by bright field microscopy (Figure S1).

### pH adjustment

The pH measurements were performed at 25°C using an Orion star A211 (Thermo Scientific^TM^) pH meter equipped with a micro pH electrode (8220BNWP, Thermo Scientific^TM^). Before each measurement, the pH meter was calibrated using a standard reference solution. The pH meter has an accuracy of 0.03 pH units at pH 4, 7, and 10, with a drift of less than 0.002 pH units per day. We used a new bottle of sodium hypochlorite for the experiments and kept it at 4°C. The measured pH of a 5% (50,000 ppm) sodium hypochlorite solution was 12.5. To ensure that the solution was stable throughout all the measurements, we systematically measured the pH of the stock hypochlorite solution. We adjusted its pH by adding different molar concentrations of HCl. The HCl concentration used for pH adjustment ranged from 10 mM to 500 mM. These adjustments resulted in hypochlorite solutions with pH values in the range of 4.5 to 12.5.

For spore disinfection, the pH-adjusted stock hypochlorite solution (50,000 ppm) was diluted 10-fold to a final concentration of 5,000 ppm. The pH of the diluted hypochlorite solutions was measured using the same method as described above. For spore disinfection, the pH of the hypochlorite solutions ranged from 7.0 to 12.0.

### Disinfection protocol and estimation of viable spore

We adopted the disinfection protocol from our earlier studies [31] and from other spore disinfection studies [10]. A schematic of the disinfection protocol is shown in Figure S2. For each measurement, spore suspensions were normalized to the same starting concentration. The spore suspension was mixed with pH-adjusted hypochlorite and vortexed for 10 sec. After 10-min incubation at 25°C, the hypochlorite was neutralized using 1% sodium thiosulfate solution. The incubation time is in line with common disinfection guidelines in hospitals, which also typically require 10 min exposure [32]. The treated spores were washed 2 times to remove the hypochlorite and thiosulfate via centrifugation at 5,000 g, for 5-min at 25°C. Finally, the spores were resuspended in deionized water. To maintain consistency, the untreated control was centrifuged similarly. The resuspended spores were serially diluted in deionized water to a final concentration of $10^{-6}$. Next, 10 $\upmu$L drops of the spore suspensions were plated onto agar plates and incubated at 37°C. For *B. cereus*, the incubation lasted for 10 hours, which was sufficient for colonies to appear but remained small enough for counting without overlapping. We imaged the plates against a dark background using a digital camera, which allowed for a clear visualization of individual colonies. Colonies were counted from appropriately diluted samples, and the dilution factor was subsequently applied to calculate colony forming units (CFU/mL). To ensure robust data, we averaged three technical replicates per biological replicate. Standard deviation was calculated across at least two biological replicates to estimate variability in the average CFU/mL. The reduction in CFU was estimated from the comparison between control and hypochlorite-treated spore samples.

### Raman spectroscopy

We used a laser tweezer Raman spectroscopy setup built around an inverted microscope (IX71, Olympus), described in more detail in previous works [33, 34]. A 785 nm Gaussian laser beam with a maximum power of 120 mW (08-NLD, Cobolt) is coupled into our microscope system via a dichroic shortpass mirror with a cutoff wavelength of 650 nm (DMSP650, Thorlabs). The beam is focused using a 60$\times$ water immersion objective with a numerical aperture of 1.2 (UPlanSApo, Olympus) and a working distance of 0.28 mm. To obtain Raman spectra, we collect the backscattered light through the same microscope objective. The light is filtered out using a 785 nm notch filter (NF785-33, Thorlabs) and then filtered using a 150 $\upmu$m diameter pinhole in the focal point of the telescope. It is then coupled into a spectrometer (Model 207, McPherson) and dispersed using 600 grooves/mm holographic grating with an 800 nm blaze. The Raman spectra are captured with a Peltier-cooled CCD detector (Newton 920N-BR-DDXW-RECR, Andor) operated at −95°C and we use Solis (Solis v4.30, Andor) software to control the detector and acquire spectra. The spectral resolution of our instrument is < 3 cm^−1^.

To record Raman spectra, we place a 10 $\upmu$L drop on a 24 $\times$ 60 mm glass coverslip (no.1, Paul Marienfeld GmbH & Co., Lauda-Königshofen, Germany). We make a vacuum grease ring (Dow Corning, Midland, MI) around the drop and place a 20 $\times$ 20 mm coverslip (no.1, Paul Marienfeld GmbH & Co., Lauda-Königshofen, Germany) on top to seal the sample to prevent evaporation. The sample is placed on a temperature-controlled micrometer stage, and the measurement temperature is maintained at 25°C throughout the experiment. We set the laser focal point at a height of 100 $\upmu$m above the glass surface to maintain a consistent baseline for the spectra with minimal influence from the coverslip [31]. To acquire Raman spectra of individual spores in this study, we trapped a spore and measured using 10 accumulations of 10 seconds each, within the spectral range of 600-1,700 cm^−1^. To prevent spore damage, irradiation was limited to below 10 J [35]. For each hypochlorite pH, Raman spectra were collected from 30 individual spores. Background spectra were acquired from the sample without trapping a spore.

For Raman measurement of NaOCl at different pH, measurements were performed in the 450-1,300 cm^−1^ range. The sample preparation and measurement were similar to those described above. A total of 10 accumulations of 10 seconds were collected, with five technical replicates for each hypochlorite pH.

### Fluorescence microscopy

Fluorescence microscopy measurements were performed using a wide-field inverted fluorescence microscope (IX70, Olympus), where the excitation is provided by a mercury lamp (U-RFL-T, Olympus) through a 470AF50 excitation filter (Omega Optics). The excitation beam is focused on the sample through a 100$\times$ microscope objective (Olympus Ach 100$\times$, 1.25 NA, oil objective). The fluorescence passes through a dichroic filter (GFP-30LP-B-OMF-ZERO, cutoff wavelength - 495) followed by a longpass emission filter (496LP) to reach the 8.9 MP color CMOS camera (CS895CU, Kiralux, Thorlabs).

To prepare a sample, spores are incubated with 100 $\upmu$M of Thioflavin T (ThT) (T3516, Sigma-Aldrich) for 10-min. A 10 $\upmu$L drop is dried on the top of a cover glass. We recorded images with 200 ms exposure time and analyzed images with Fiji [36]. To assess spore fluorescence intensity, RGB images were separated into individual red, green, and blue channels. The green channel, which corresponds to ThT fluorescence, was selected for further analysis. Intensity measurements were obtained from the core of each spore using the multi-point tool in Fiji image analysis software.

### Tracking cell viability after hypochlorite treatment

To confirm spore germination and cell division after disinfection at pH 11.8, we incubated an aliquot of hypochlorite treated spore suspensions in Tryptic Soy Broth (TSB) (Bacto^TM^, BD) at 37°C for 4 hours, allowing the spores to germinate and initiate cell division. The spores were then imaged in brightfield mode using the microscope in our Raman setup described above.

### Data analysis

To process our Raman spectra, we used an open-source MATLAB (MATLAB2022b, Mathworks) script provided by the Vibrational Spectroscopy Core Facility at Umeå University [37]. It baseline-corrects the spectra using an asymmetrical least squares algorithm [38] and with the algorithm parameters set to $\lambda$ = 10^6^ and p = 10^−3^. To test the bimodal distribution of the data with spores releasing the calcium chelate DPA (CaDPA), we used Warren Sarle’s bimodality coefficient calculated using a user script in MATLAB [39].

We verified the statistical significance of results for CFU counts with an ANOVA test with multiple comparisons (Šídák’s multiple comparisons tests), using Prism (Prism 10.3, GraphPad).

We used Origin 2024 (OriginLab) to plot and fit functions to the data. To model spore viability as a function of hypochlorite pH, we used a biphasic response function,

5 $$\begin{aligned} y = A_1 + (A_2 - A_1) \left[ \frac{f}{1 + 10^{(k_1 - x) h_1}} + \frac{1 - f}{1 + 10^{(k_2 - x)h_2}} \right] \end{aligned}$$

Here, *x* is the pH of the hypochlorite solution, *A*_1_ and *A*_2_ are the minimum and maximum asymptotes of the function, *h*_1_ and *h*_2_ are the slopes of the transitions, *k*_1_ and *k*_2_ are the midpoints of the transitions, and *f* is the fraction representing the relative amplitude of the two phases.

We used the relationship between the pK_a_ and pH [40] to fit the transition between the HOCl and OCl^-^ states, which is described by

6 $$\begin{aligned} y = \frac{10^{(x - pK_{a})}}{1 + 10^{(x - pK_{a})}} \end{aligned}$$

Here, *x* is the pH of the hypochlorite solution. The derivation of the above equation from the Henderson-Hasselbalch equation is described in the supplementary information.

To fit the change in pH of sodium hypochlorite solution over time, we used an exponential function,

7 $$\begin{aligned} y = y_0 + E_1 \exp \left( \frac{-t}{t_1}\right) \end{aligned}$$

Here, *y*_0_ is the offset, *E*_1_ is the amplitude, *t* is the incubation time, and *t*_1_ is the time constant.

## Results and discussion

### Hypochlorite’s sporicidal efficiency changes sharply between pH 9.5 and 11.0

Despite hypochlorite being one of the most widely used disinfectants globally [6], published data on its sporicidal efficiency remains inconsistent. A primary reason for this inconsistency is the insufficient reporting on the pH conditions under which disinfection is performed, as shown in Table 1. To address this issue, we systematically adjusted the pH of a hypochlorite stock solution, reducing it from its initial pH of 12.0 to a neutral pH of 7.0 using HCl. Spores were treated with hypochlorite at different pH levels and their viability was quantified by determining CFUs. Representative agar plate images are shown in Figures S3 and S4.

**Fig. 1:**
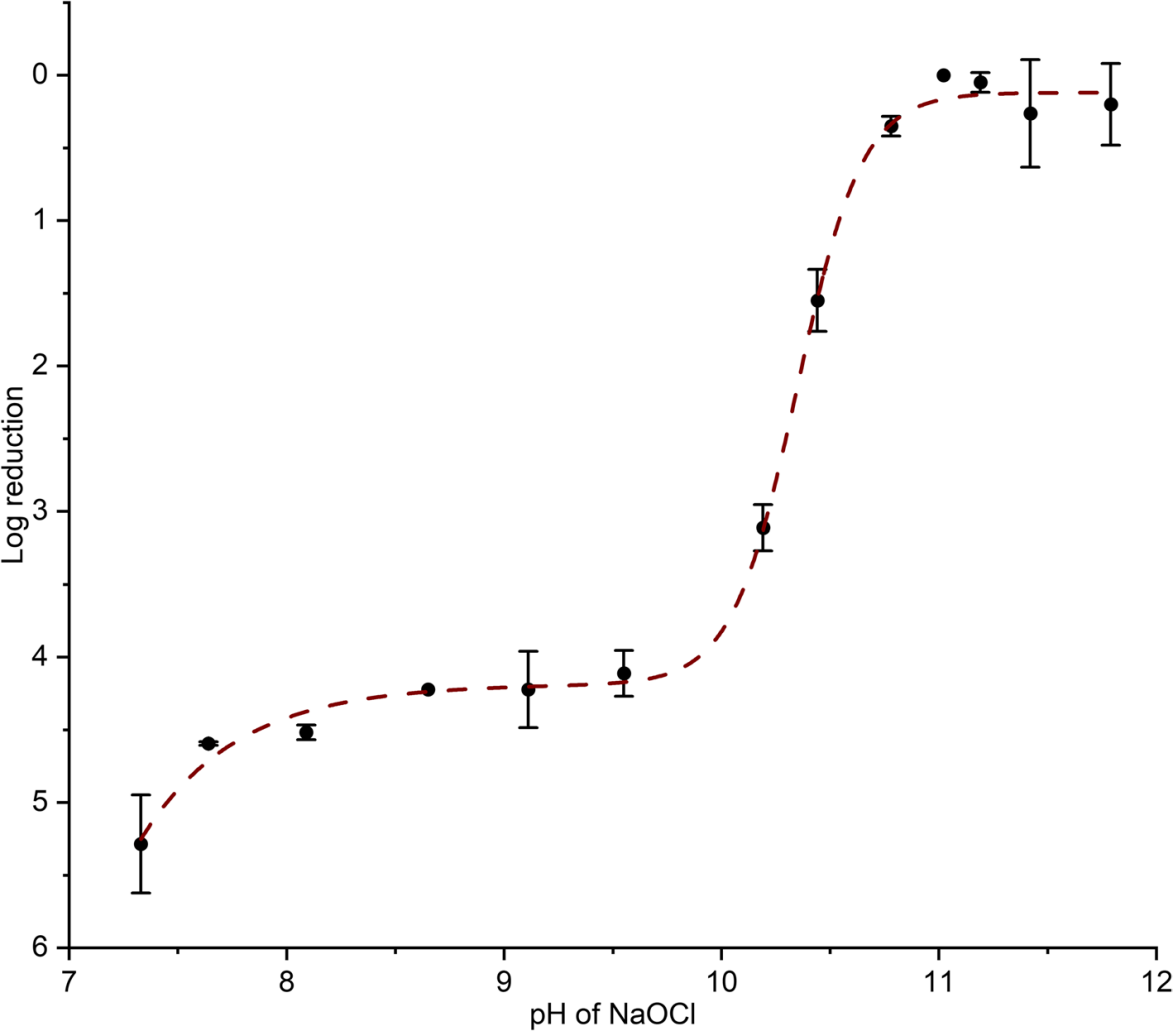
Log reduction of *B. cereus* spore viability as a function of the pH of sodium hypochlorite solutions (black filled circles). At high pH, hypochlorite has a negligible effect on spore viability. Spore inactivation increases sharply between pH 11 and 9.5, with more than 4-log reductions in CFUs. A further decrease in pH to 7.5 increases the sporicidal activity by an additional 1-log. The brown dashed line is a fit of Eq. 5 (R^2^ = 0.99), showing a typical S-shaped dose-response curve. The error bars represent the SDs calculated from at least two biological replicates, each consisting of 3 technical replicates

Our findings reveal that the 5,000 ppm hypochlorite solutions are ineffective at killing spores when the pH is between 11.0 and 12.0, as shown in Fig. 1 and Figure S5, with no reduction in viability at pH>11.0. This result was further supported by bright field imaging of spores incubated in nutrient media, which showed that most spores germinated and developed into vegetative cells (Figure S6).

We observed a significant shift in sporicidal efficacy between pH 11.0 and 9.5, where the CFU count decreased from 1$\times 10^{7}$ to 8.0$\times 10^{2}$ CFU/mL as the pH decreased, representing a 4.1-log reduction (Fig. 1). At a pH of 7.3, the viability further decreased to 1.0$\times 10^{2}$, indicating an additional 0.9-log reduction. To evaluate the response and determine the midpoint transition pH (10.3±0.05) we applied a biphasic response function (Eq. 5). Notably, previous studies looking at spore decontamination have not identified a critical shift within the pH range of 9.5 - 11.0, as they have predominantly focused on comparisons between physiological and alkaline pH conditions without specifically addressing this transition. Since an improvement in sporicidal ability has been observed at a lower pH for different species, as highlighted in Table 1, a similar transition in sporicidal efficiency is expected in other *Bacillus* and even *Clostridium* species due to general similarity in bacterial spore structure. However, factors such as exosporium and differences in coat thickness and composition are likely to play a significant role in the pH range of the transition.

### pH-dependent changes in hypochlorite chemistry and shelf life

To investigate the underlying causes of the sporicidal effect, we assessed changes in hypochlorite at the transition pH. Sodium hypochlorite exists in two ionic forms, ClO^-^ at high pH and HOCl at neutral pH [8, 41]. At acidic pH values ($<5$) hypochlorite hydrolyzes into chlorine gas and water. The shift in the sporicidal efficacy of hypochlorite at physiological pH is typically attributed to this change in ionic form. The reported pKa of hypochlorite varies and generally falls into two categories: around $\sim$7.5, first reported by Morris et al., [41] based on titration data, and 7.93, calculated by Chemicalize for the hypochlorite ion at 25°C [42]. To account for any discrepancies between these reported pKa values, we used Raman spectroscopy to identify the transition between the ionic states by monitoring the change of the 713 and 726 cm^−1^ peaks corresponding to the two ionic forms of hypochlorite, ClO^-^ and HOCl [43]. Raman spectroscopy is an established technique for observing such ionic state transitions [44–46].

**Fig. 2:**
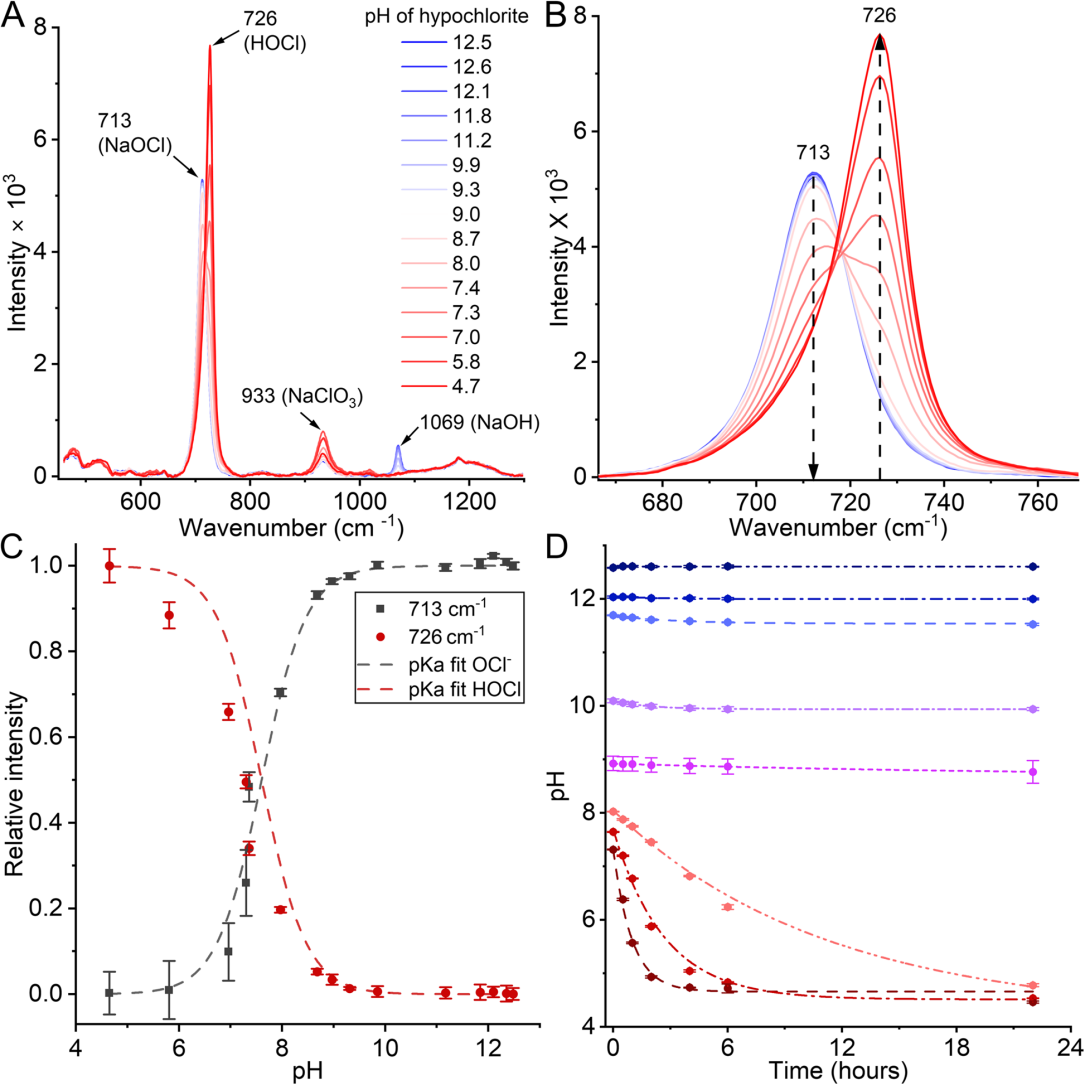
Raman spectra of hypochlorite solutions at varying pH levels. **A** Raman peak intensities of hypochlorite across pH 4.7–12.5, with key peaks at 713 cm^−1^ (NaOCl), 726 cm^−1^ (HOCl), 933 cm^−1^ (NaClO_3_), and 1069 cm^−1^ (NaOH). The blue-to-red gradient represents pH from high to low. Chlorine-related components form as shown in Eqs. 1-3, with NaOH acting as a stabilizer. **B** Raman spectrum of the characteristic peaks of the hypochlorite ionic forms show the 713 cm^−1^ peak (ClO^-^) dominating at high pH, transitioning to 726 cm^−1^ (HOCl) between pH 7.0–9.0. **C** Normalized intensities vs pH of the peaks 713 cm^−1^ (black square) and 726 cm^−1^ (red dots), fitted (dashed lines) using Eq. 6 (R^2^ = 0.99). From these fittings, the pK_a_ is estimated as 7.6±0.05. **D** pH stability of 50,000 ppm hypochlorite solutions over time at 25°C. Above pH 8.9, hypochlorite remains stable over time, while solutions with pH <8.9 exhibit exponential pH decline. Dashed lines represent exponential fits (Eq. 7), yielding time constants (*t*_1_) of 11.2±1.3, 2.7±0.2, and 0.9±0.1 h for pH 8.0, 7.7, and 7.3, respectively

The Raman spectrum of our solution of sodium hypochlorite revealed several major peaks that were sensitive to changes in solution pH. These peaks are specifically at 713, 726, 933, and 1069 cm^−1^, as shown in Fig. 2A. Our Raman data also indicate that the peak at 713 cm^−1^ is associated with high pH, whereas the peak at 726 cm^−1^ corresponds to low pH, as reported in the literature (Fig. 2B). By fitting a logarithmic curve (Eq. 6) to the normalized peak intensities, we estimated the pKa to be 7.6±0.05 (Fig. 2C), which aligns with the values previously reported by Morris et al. [41].

The other two peaks observed in the Raman spectra do not directly originate from the hypochlorite molecule. The peak at 1069 cm^−1^, which significantly decreases in intensity at pH<9, is attributed to NaOH (sodium hydroxide) [47], a component included in stock sodium hypochlorite solutions to maintain their stability. As shown in Figure S7, this peak can be seen in pure aqueous NaOH and is very prominent in anhydrous NaOH powder. Additionally, the spectra show a distinct peak at 933 cm^−1^, which we attribute to an intermediate chlorine species resulting from the decomposition of NaOCl. Hypochlorite is known to decompose into chlorate ion (ClO_3_^-^) as a function of pH [48, 49]. Notably, the intensity of the 933 cm^−1^ peak increases with decreasing pH, reaching a maximum around pH 6.0, before diminishing at lower pH values (around 4.0), where hypochlorite decomposes into water and chlorine. Figure S8 shows a peak at 933 cm^−1^ in the Raman spectrum of sodium chlorate, a finding further supported by existing literature [50, 51]. Therefore, we propose that the peak at 933 cm^−1^ corresponds to NaClO_3_, an intermediate species generated during sodium hypochlorite decomposition. The decreasing intensity of this peak at lower pH values aligns with the continued decomposition of the compound under acidic conditions.

To further investigate the proposed mechanism of action, which suggests that the protonated ionic form, HOCl, is responsible for the sporicidal effect of hypochlorite [11, 52], we adjusted the pH to achieve specific concentrations of HOCl. We observed a dose-response behavior (Figure S9), where no sporicidal effect was observed below a threshold concentration of 1.9 ppm. However, in the concentration range of 1.9 to 12.8 ppm, spore viability followed a logarithmic relationship, with a 1-log reduction in viable spores for every 3.5 ppm increase in HOCl. At concentrations above 12.8 ppm HOCl, spore inactivation reached a plateau, with a minimal increase in efficacy observed from 12.8 to 55 ppm.

Although a lower pH increases the sporicidal efficacy of hypochlorite, it can negatively impact the shelf life of hypochlorite solution. To evaluate the shelf life of the hypochlorite solution at different pH values, we prepared fresh hypochlorite solutions at pH values ranging from 12.5 - 7.3 and incubated them at 25°C. The pH of each solution was measured at regular intervals. As shown in Fig. 2D, no significant pH change was observed after 24 hours for solutions with an initial pH of 8.9 or higher. However, a rapid exponential decrease in pH was observed for solutions with an initial pH of 8.0 and lower. These findings are consistent with the results of Adam & Gordon [14], who reported an accelerated pH decrease at pH values <10, although over a longer timescale of weeks and months.

After the initial reduction, the pH of these solutions plateaued at about pH 4.5, corresponding to the pKa of sodium chlorate salt at 4.6. This plateau indicates the formation of NaClO_3_ (Eq. 3) over time, which was validated by measuring Raman spectra of a pH 7.3 hypochlorite solution at different time intervals (Figure S10). Our data shows that the pH reduction rate and, consequently, the reaction rate, was inversely correlated to the starting pH, with lower pH solutions reaching the plateau faster than those with higher pH levels (Fig. 2D). Exponential fitting of the data (Eq. 7) yielded a time constant (*t*_1_) of 0.9±0.1 h for pH 7.3 and 11.2±1.3 h for pH 8.0. These pH changes over time suggest that at pH 8.0 and below, HOCl rapidly dissociates, producing ClO_3_^-^ and H^+^ (Eq. 3). This process results in the progressive accumulation of H^+^ ions, which gradually lowers the pH of the solution. Additionally, the rate of pH reduction is strongly influenced by the initial concentration of hypochlorite in the solution.

To investigate the effect of initial concentration on the pH reduction rate, we compared a 50,000 ppm with a 5,000 ppm hypochlorite solution at different starting pH levels. The results show a significant difference in kinetics, with the lower concentration exhibiting a 100-fold slower rate of pH reduction (Figure S11). These findings suggest that diluting a pH 9 hypochlorite solution improves its shelf life. This observation is consistent with previous studies, which have reported decreased stability of hypochlorite at lower pH levels and its strong dependence on the initial concentration [48].

We conclude that effective spore decontamination with hypochlorite requires careful pH optimization to ensure maximal sporicidal effect while maintaining the acceptable shelf life of the disinfection solution. Based on our findings, we recommend maintaining the pH of the hypochlorite solution within a range of 9.0 to 9.5, as it offers an optimal balance between stability and biocidal effect. Importantly, dilution of hypochlorite in water is also known to reduce its pH. However, at a concentration of 5,000 ppm, the pH remains at 11.9 (Figure S12). This indicates that the solution is unsuitable for spore disinfection without proper pH adjustment. We use HCl for adjustment of hypochlorite pH, however other acid e.g., acetic acid is also recommended by US Environmental Protection Agency (EPA) for this purpose [53, 54]. Importantly, care must be taken while mixing hypochlorite with acid to avert unwanted incidents due to chlorine generation [55].

### Low pH hypochlorite increases spore permeability and CaDPA release

**Fig. 3:**
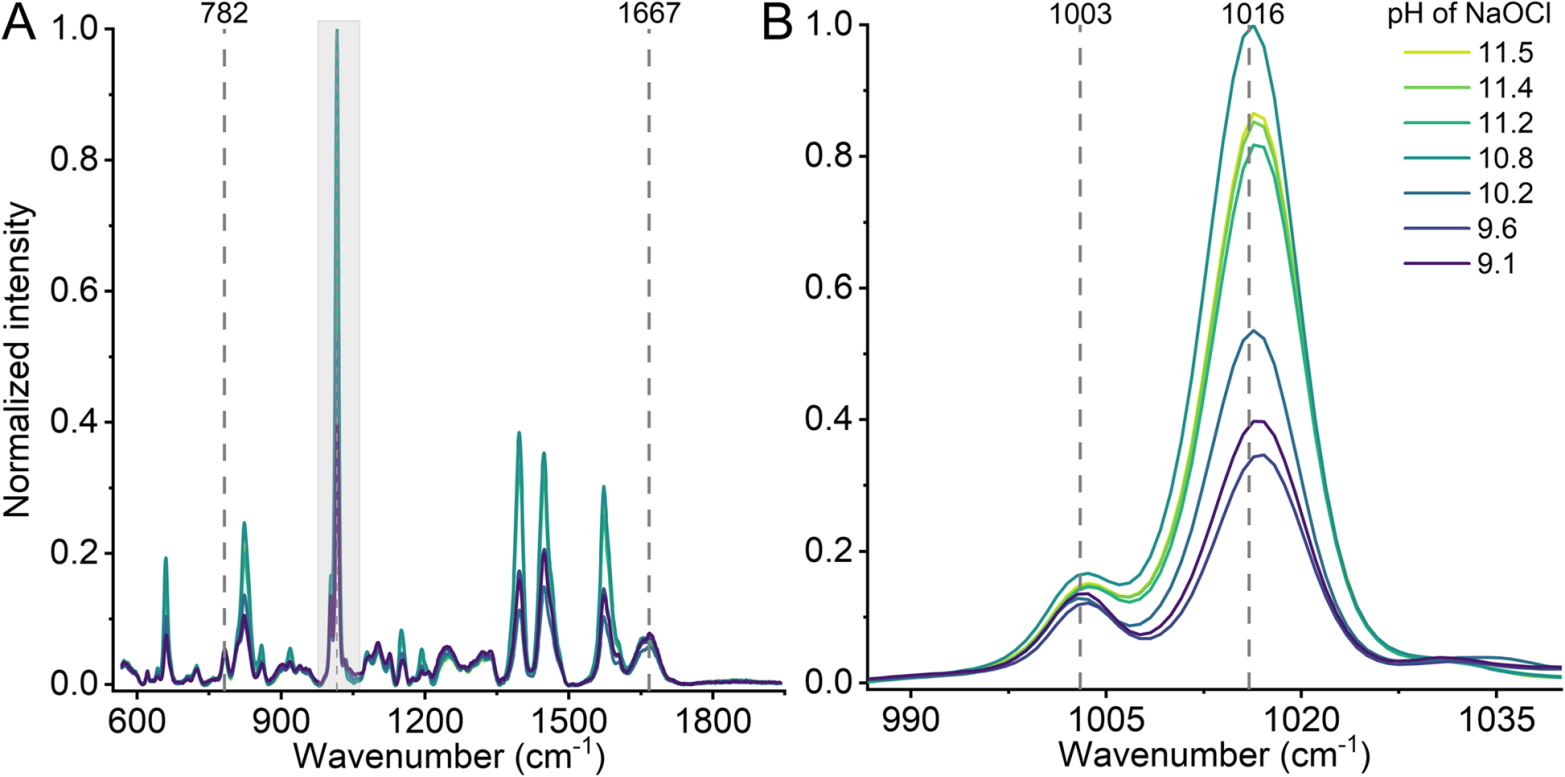
Raman spectra of spores exposed to hypochlorite of varying pH. **A** Characteristic peaks associated with DNA (782 cm^−1^), CaDPA (1016 cm^−1^), and protein (Amide I, 1667 cm^−1^) are observed. The intensity of these peaks shifts with changes in hypochlorite pH. **B** The 1016 cm^−1^ peak, attributed to CaDPA, is reduced as pH decreases from 11.5 to 9.1. This reduction indicates a loss of CaDPA due to hypochlorite activity. Note that the phenylalanine peak at 1,003 cm^−1^ remains unaffected. A detailed analysis of peak intensity distribution is provided in supplementary Figure S13

To investigate the underlying chemical changes in spores after exposure to sodium hypochlorite, we performed Raman spectroscopy on the treated samples. This analysis provides insight into NaOCl’s sporicidal mechanisms and also examines the influence of pH on spore integrity. Figure 3 presents the Raman spectra of spores, while Table 2 details the assignment of the different Raman peaks [56]. The change of intensity in Raman peaks associated with DNA, phenylalanine, CaDPA, and Amide I as a function of hypochlorite pH is shown in Figure S13. Notably, the major changes in the spectra occur at the 1016 cm^−1^ Raman peak associated with CaDPA. As shown in Fig. 3B and Figure S13C, the intensity of this peak decreases with a reduction in hypochlorite pH. Specifically, the intensity remains relatively constant at pH values up to 10.8.

**Table 2:** Peak locations and assignments of relevant spore Raman peaks

Peak location (cm^−1^)	Assignment
782	DNA O-P-O stretch
1,003	Phenylalanine ring breathing
1,016	CaDPA ring breathing
1,667	Amide I (protein backbone)

A further decrease in pH resulted in a 60% reduction in the intensity of the CaDPA peak, suggesting the release of CaDPA at lower hypochlorite pH levels. A striking observation is the bimodal intensity distribution. Approximately 60% of the spore population undergoes complete CaDPA release, a phenomenon likely attributable to the enhanced permeability of the spore core at reduced pH. Figure S13 also shows how the intensity of other characteristic Raman peaks from spore changes as a function of hypochlorite pH. For example, the DNA and protein peaks remain unaffected by pH changes. This observation aligns with our previous results, demonstrating that hypochlorite induces changes in the spore coat and inner membrane, as visualized by TEM imaging [31]. Furthermore, prior de-coating of spores or mutations in genes essential for coat assembly significantly increased the sensitivity of spores towards hypochlorite [10]. This finding suggests that the damage induced by hypochlorite treatment is primarily associated with alterations in inner membrane structure. Lowering the hypochlorite pH may amplify this damage, likely due to the formation of nonionic HOCl.

Finally, the absence of changes in the 782 cm^−1^ peak associated with DNA suggests that the DNA structure remains intact and that the openings formed are insufficient to allow DNA to escape from the core. The lack of *B. cereus* DNA damage upon hypochlorite treatment is consistent with the previous observation reported by Young and Setlow using *B. subtilis* spores [10].

**Fig. 4:**
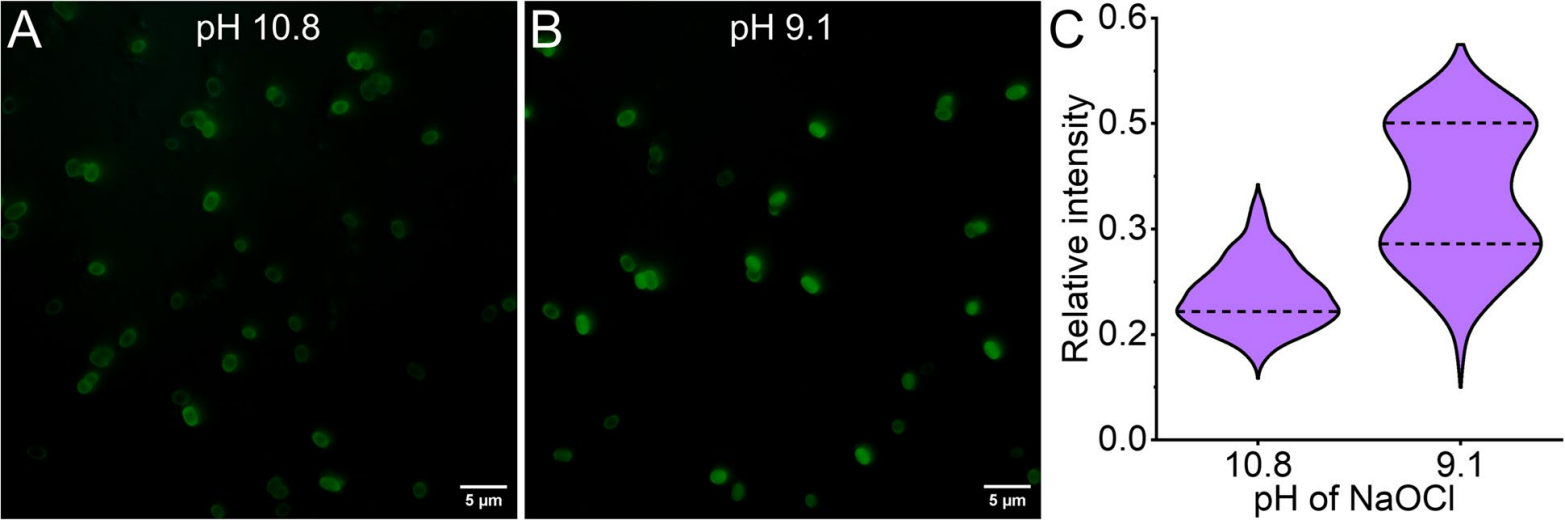
Fluorescence microscopy of NaOCl-treated spores stained with ThT. **A** High-pH treatment (pH 10.8) preserves spore viability, preventing ThT penetration, as observed with the pale outlines. **B** In contrast, decontaminated spores within the transition range (pH 9.1) show increased permeability to ThT, leading to ThT uptake in up to 50% of the spore population. **C** Distribution of fluorescence intensity from the core of spores at pH 10.8 (n = 498) and pH 9.1 (n = 495). The intensity is normalized to the maximum fluorescence intensity. The dashed lines represent the modes of the distribution. Bimodal intensity distribution in pH 9.1 indicates differential permeability of the spore upon hypochlorite treatment in pH 9.1

To further investigate the mode of action of NaOCl as a function of pH, we used ThT as a spore permeability marker and employed fluorescence imaging for detection. ThT is a benzothiazole dye widely used as a marker for detecting amyloid aggregates. However, in several instances, ThT has been used to detect bacterial cells and spores [57–59]. After disinfection, the spores are incubated with ThT and imaged using a bright-field fluorescence microscope, as shown in Fig. 4. We observed that reducing the pH of the NaOCl solution from 10.8 to 9.1 resulted in much brighter spores. Figure 4C shows the ThT fluorescence intensity distribution of the spore core. At pH 10.8, the fluorescence intensity shows an unimodal distribution, with the peak centered around 0.22. However, at pH 9.1 the bimodal distribution is observed with peaks centered at 0.27 and 0.50, respectively. This increased ThT intensity in many spores indicates greater permeability, allowing more ThT molecules to enter the spore. Previous studies using ThT and spores have shown that ThT can differentiate between spores undergoing heat treatment, with the internalization of ThT in heat-treated spores attributed to damaged spore coat structure [59]. The changes in spore permeability and viability correlate with the pH shift between 10.8 and 9.1; however, the number of permeable spores (50%) is much lower than the expected fraction of killed spores (99.99%). Hence, the higher permeability of spores is an after-effect of decontamination rather than a determining factor in their inactivation.

### Stable and effective hypochlorite solutions require an optimal pH

**Fig. 5:**
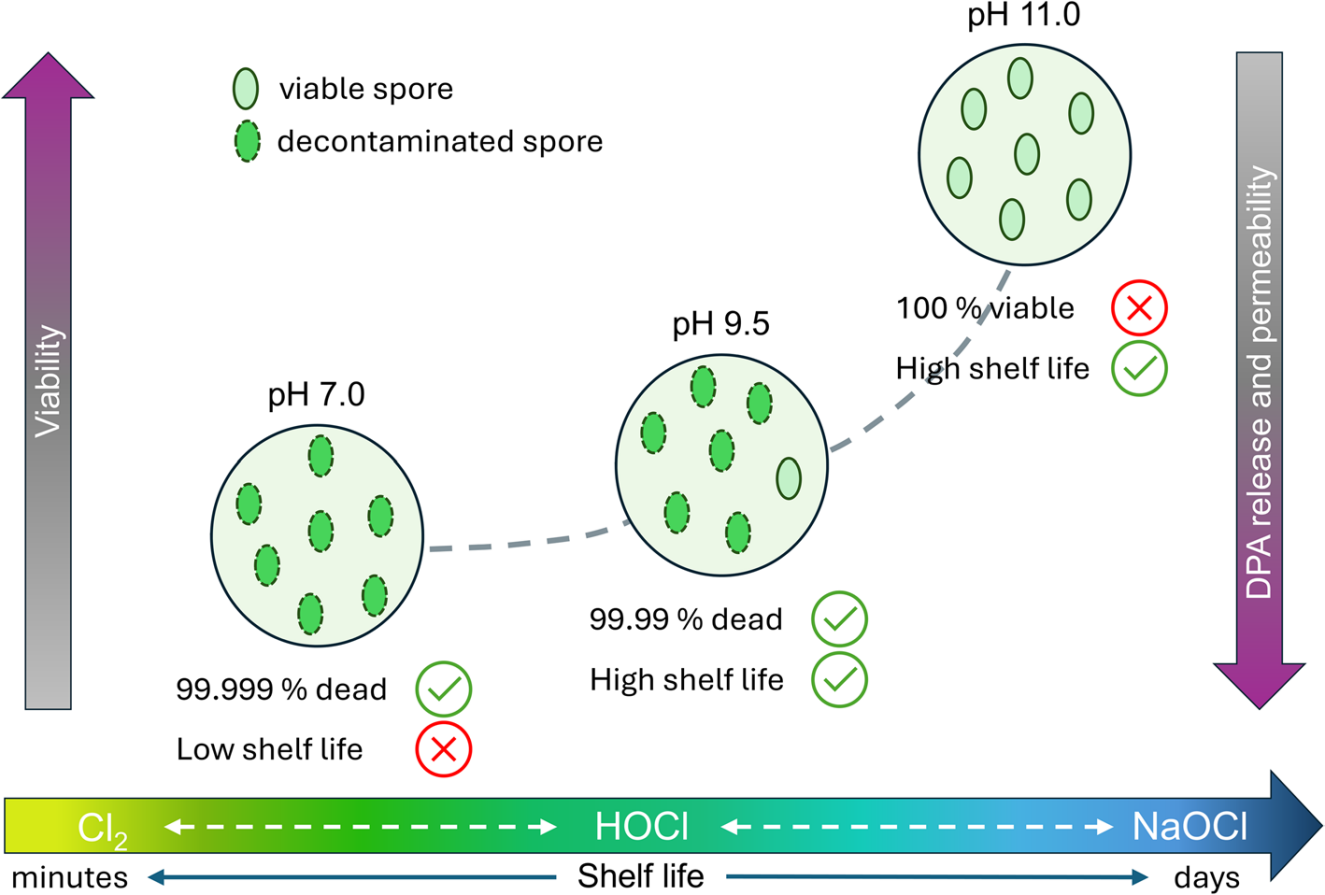
Schematic illustrating the interplay between hypochlorite treatment, spore viability, and solution shelf life. Under acidic conditions, spores are rapidly inactivated, but the stability of the solution is weak, resulting in a short shelf life. On the other hand, at high pH, the hypochlorite solution is stable but ineffective against spores. An intermediate pH of 9.5 offers a compromise, ensuring effective spore decontamination while preserving the stability and shelf life of hypochlorite

The effectiveness of hypochlorite for decontaminating bacterial spores is intricately linked to its pH and shelf life. While a reduction in pH improves its sporicidal efficiency, it comes with the cost of its chemical stability. The existing neutral pH hypochlorite commercial products have to adapt to the issue by storing a stabilized solution that is mixed with a neutralizing agent immediately before use (for example, PCS 250 Disinfectant Cleaner, PCS), or have a product that needs to be used soon after being opened. Establishing an optimal balance between pH and shelf life is crucial to ensure the stability and efficacy of this disinfecting agent without the added complexity of mixing from stable precursors. The schematic in Fig. 5 shows the relation between different parameters concerning pH, shelf life and sporicidal efficiency of hypochlorite. The reduction in pH from 11 to 9.5 shows 4-log reduction in spore viability (99.99% dead spores), without compromising the shelf life of hypochlorite. Further reduction in pH to 7 improves the sporicidal efficiency by 0.9 log. However, at this pH, the shelf life at room temperature falls within only a few hours, which means it needs to be used immediately after pH adjustment.

Diluting bleach does improve its shelf life, as the main contributing decomposition reactions are second-order (dominant at pH $>9$) and third-order (dominant at pH 5–9), and thus, diluting hypochlorite solutions will greatly reduce the decomposition rate [14]. This implies that storage of a 5,000 ppm pH adjusted (pH 9.0 - 9.5) hypochlorite solution is preferred over 50,000 ppm stock hypochlorite solution. However, diluted hypochlorite is more sensitive to being neutralized under practical usage conditions due to the presence of various organic contaminants on surfaces that react with it. For example, Byun et al. compared the efficacy of sodium hypochlorite against *Salmonella* Enteritidis planktonic cells in clean and dirty conditions and showed a 4-log decrease in disinfection efficacy in dirty conditions compared to clean [60]. Similar considerations about the level of organic load on the surface need to be made when using low ppm pH-adjusted hypochlorite formulations to avoid solution neutralization below the disinfection thresholds.

## Conclusion

Sodium hypochlorite is one the most widely used disinfectants in the world and is often employed for cleaning spore-contaminated surfaces. However, the efficacy against bacterial spores is highly pH-dependent. Our study shows that a pH of 9.5 optimizes the sporicidal activity of hypochlorite against *B. cereus* spores, achieving a 4-log reduction in viability. This pH not only maximizes sporicidal activity but also supports the development of pre-made sporicidal formulations with a reasonable shelf life, making hypochlorite more practical for widespread disinfection applications. These findings are important for the food industry and healthcare institutions, where spore contamination poses persistent challenges. By identifying the optimal pH for hypochlorite-based sporicidal formulations, this study provides valuable insights for developing more effective, stable and sustainable disinfection strategies. Altogether, the results of this study demonstrate how to improve protocols for controlling spore-forming bacteria, ultimately enhancing safety and efficiency in high-risk settings.

## Supplementary Information

Supplementary Material 1.

## Data Availability

The datasets supporting the conclusions of this article are included within the article and its additional files.

## Abbreviations

CaDPA: Calcium dipicolinic acid
CRA: Chlorine releasing agent
CFU: Colony forming units
LTRS: Laser tweezer Raman spectroscopy
ThT: Thioflavin T
TSA: Tryptic soy agar
