# Supplementary material for: Boosting hypochlorite’s disinfection power through pH modulation
Source: BMC Microbiol. 2025 Feb 28;25:101. doi: 10.1186/s12866-025-03831-w (PMC11869716; doi:10.1186/s12866-025-03831-w)
Supplement: Supplementary file 1 — Supplementary Material 1. [file 12866_2025_3831_MOESM1_ESM.pdf]

Supporting information

Boosting Hypochlorite's Disinfection Power through pH Modulation

Timir Baran Sil[1], Dmitry Malyshev[1], Marina Aspholm[2], and Magnus Andersson[1,3]*

[1]Department of Physics, Umeå University, SE-901 87 Umeå (Sweden)

[2]Department of Paraclinical Sciences, Norwegian University of Life Sciences, Ås 1432 (Norway)

[3]Umeå Centre for Microbial Research (UCMR), Umeå University, Umeå 90187 (Sweden)

*Corresponding author: magnus.andersson@umu.se

Table of Contents

Figure S1: Bright field microscopy image of purified *B. cereus* spore suspension.

Figure S2: Schematic of disinfection protocol using hypochlorite at different pH.

Figure S3: Agar plate drop counts of the hypochlorite disinfection of *B. cereus* spores.

Figure S4: Disinfection of *B. cereus* spores at lower pH of hypochlorite.

Figure S5: Comparison of CFU count between control spores and sodium hypochlorite treated spore without pH adjustment (pH = 11.8).

Figure S6: Spore germination and cell division after disinfection with sodium hypochlorite (pH = 11.8).

Figure S7: The evolution of the 1069 cm^{-1} Raman band with pH.

Figure S8: The evolution of the 933 cm^{-1} Raman band with pH.

Figure S9: Viability of spores in relation to the concentration of the HOCl form of hypochlorite.

Figure S10: Raman spectra of sodium hypochlorite (pH 7.3) at different time intervals.

Figure S11: The change in pH of 5,000 ppm hypochlorite solution over time.

Figure S12: The pH of hypochlorite solution as a function of dilution in water.

Figure S13: Intensity of characteristic Raman bands after disinfection with hypochlorite of different pH.

Derivation of the relationship between pH and pKa to determine pKa from Raman intensity.

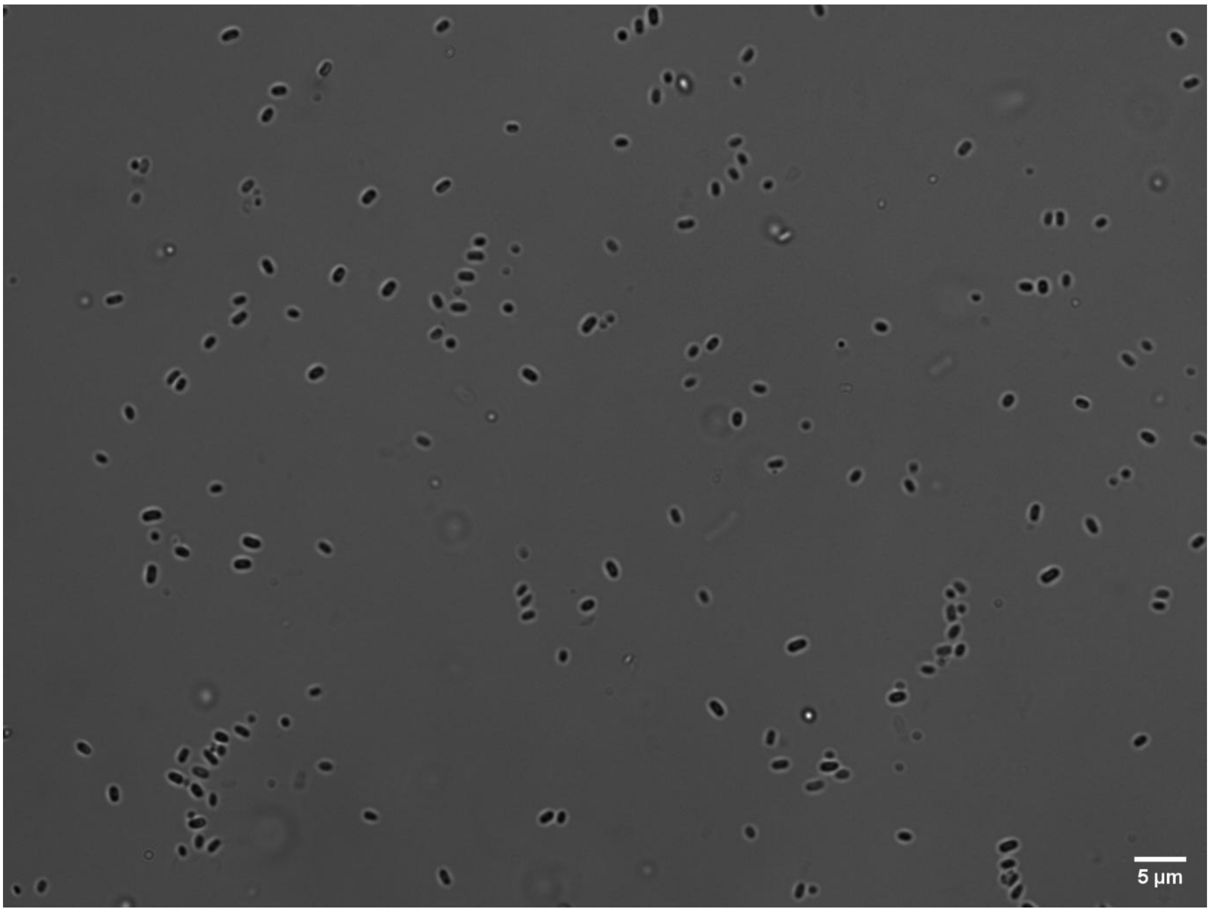

Figure S1. Bright field microscopy image of purified *B. cereus* spore suspension. The image is acquired using same inverted microscope which is used for Raman measurements. The detail about the microscope is provided in material and method section of the manuscript.

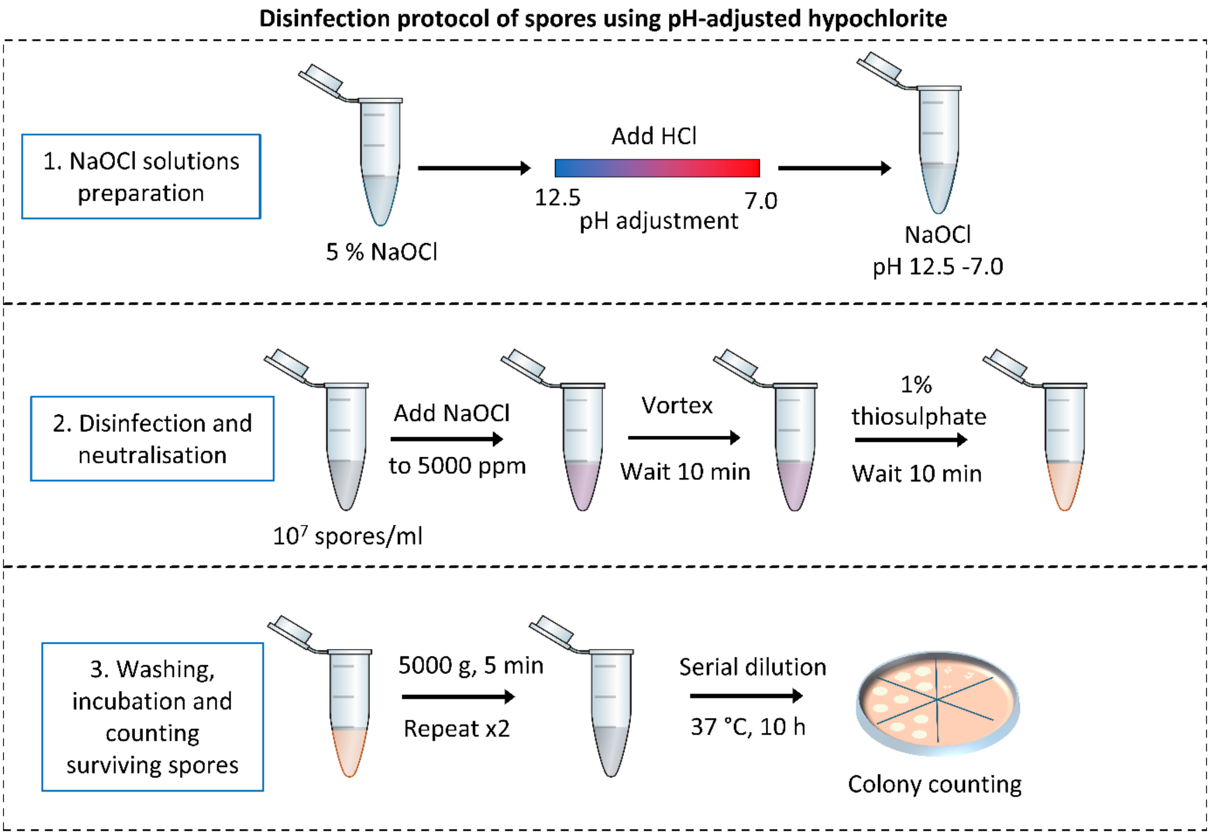

Figure S2. Disinfection protocol for bacterial spores using pH-adjusted hypochlorite. The protocol consists of three steps: (1) Preparation of NaOCl solution, where 5% (50,000 ppm) NaOCl is adjusted from pH 12.5 to 7.0 using HCl; (2) Disinfection and neutralization, where 5,000 ppm NaOCl is added to a spore suspension (10^7 spores/ml), followed by 10 minutes of vortexing and neutralization with 1% sodium thiosulfate; (3) Washing, incubation, and counting, where spores are washed via centrifugation, serially diluted, and incubated at 37°C for 10 hours for colony counting.

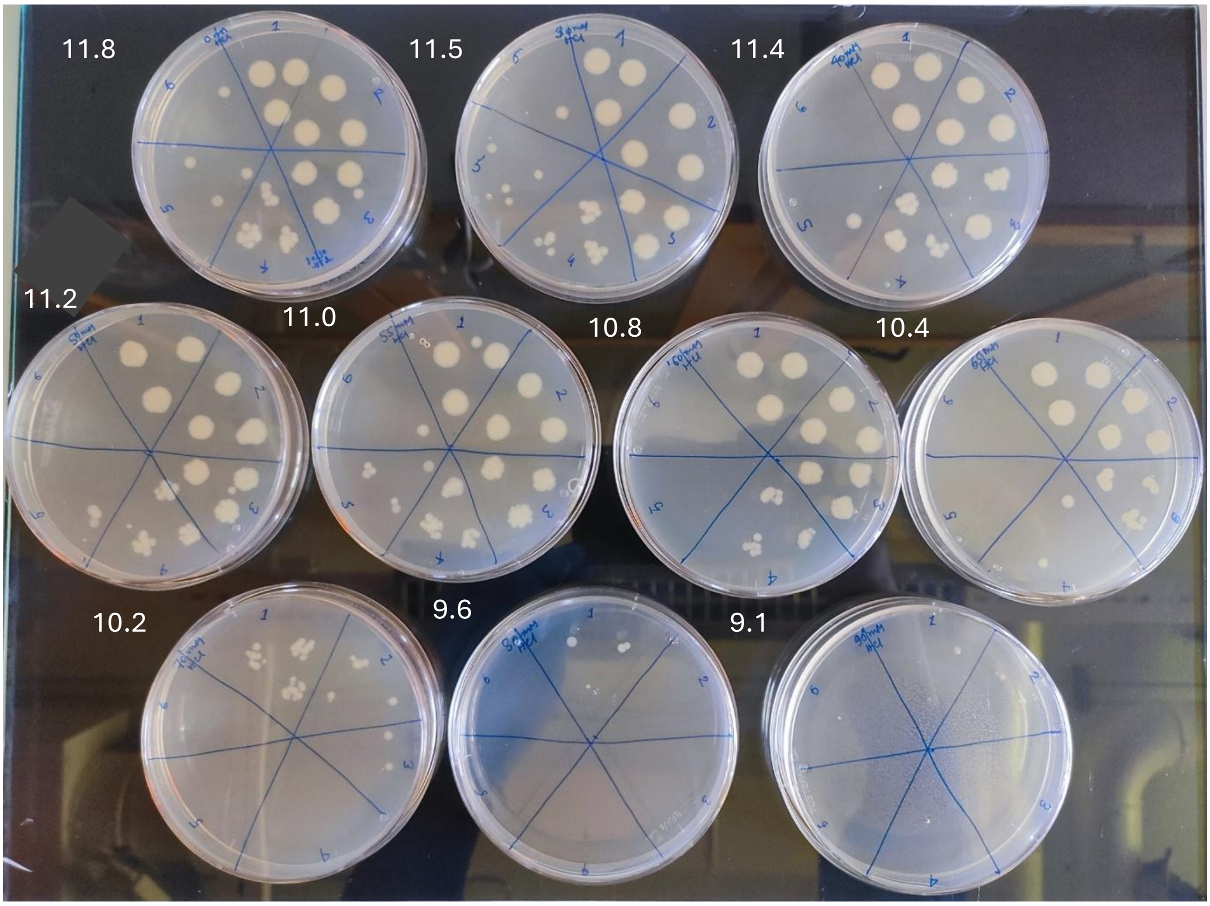

pH	11.8	11.5	11.4	11.2	11.0	10.8	10.4	10.2	9.6	9.1
CFU/mL	1×10^7	1×10^7	1×10^7	1×10^7	1×10^7	5×10^6	4×10^5	1×10^4	$<1\times10^3$	$<1\times10^3$

Figure S3. A replicate set of the hypochlorite disinfection of *B. cereus* spores depending on pH. Plates were labelled based on the concentration of HCl in the solution. The table below shows the recoded pH. As can be seen, while the initial addition of HCl and pH changes did not affect sporicidal efficiency, a transition occurs after pH 11. The pH values are indicated on the left side of the plates.

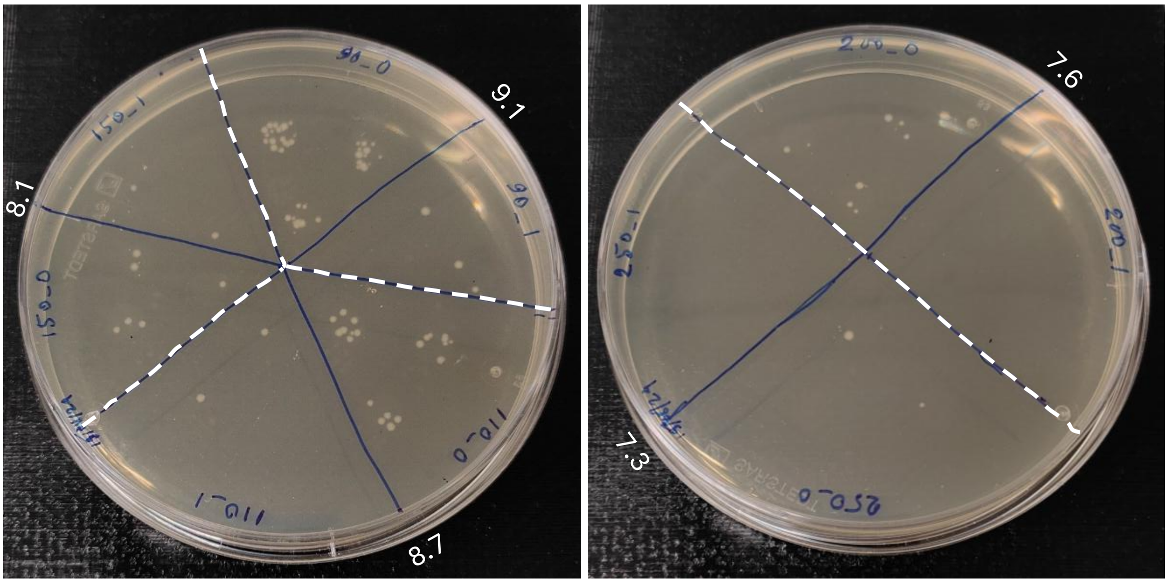

pH	9.1	8.1	8.6	7.6	7.3
CFU/mL	9×10^{2}	6×10^{2}	2×10^{2}	2×10^{2}	1×10^{2}

Figure S4. Disinfection of *B. cereus* spores at lower pH values. Due to CFU counts being $<1\times10^{3,}$ we use non-diluted drops, indicated as dilution 0. pH values are indicated.

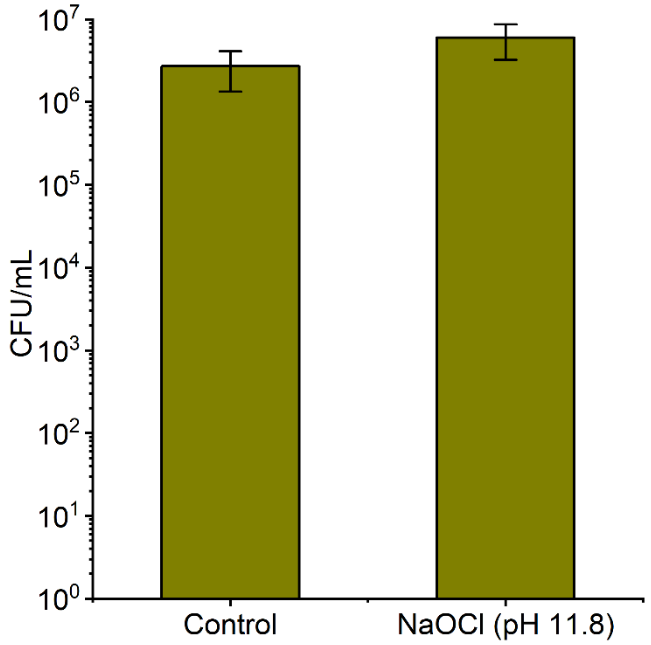

Figure S5. Comparison of CFU counts between untreated control spores and sodium hypochlorite treated spores without pH adjustment (pH = 11.8). After hypochlorite treatment (Control spores are treated with deionized water), the surviving spores are counted according to the protocol shown in Figure S1. The values reported here are averages of two or more biological replicates, and error bars are standard deviations. Each biological replicate consists of three technical replicates.

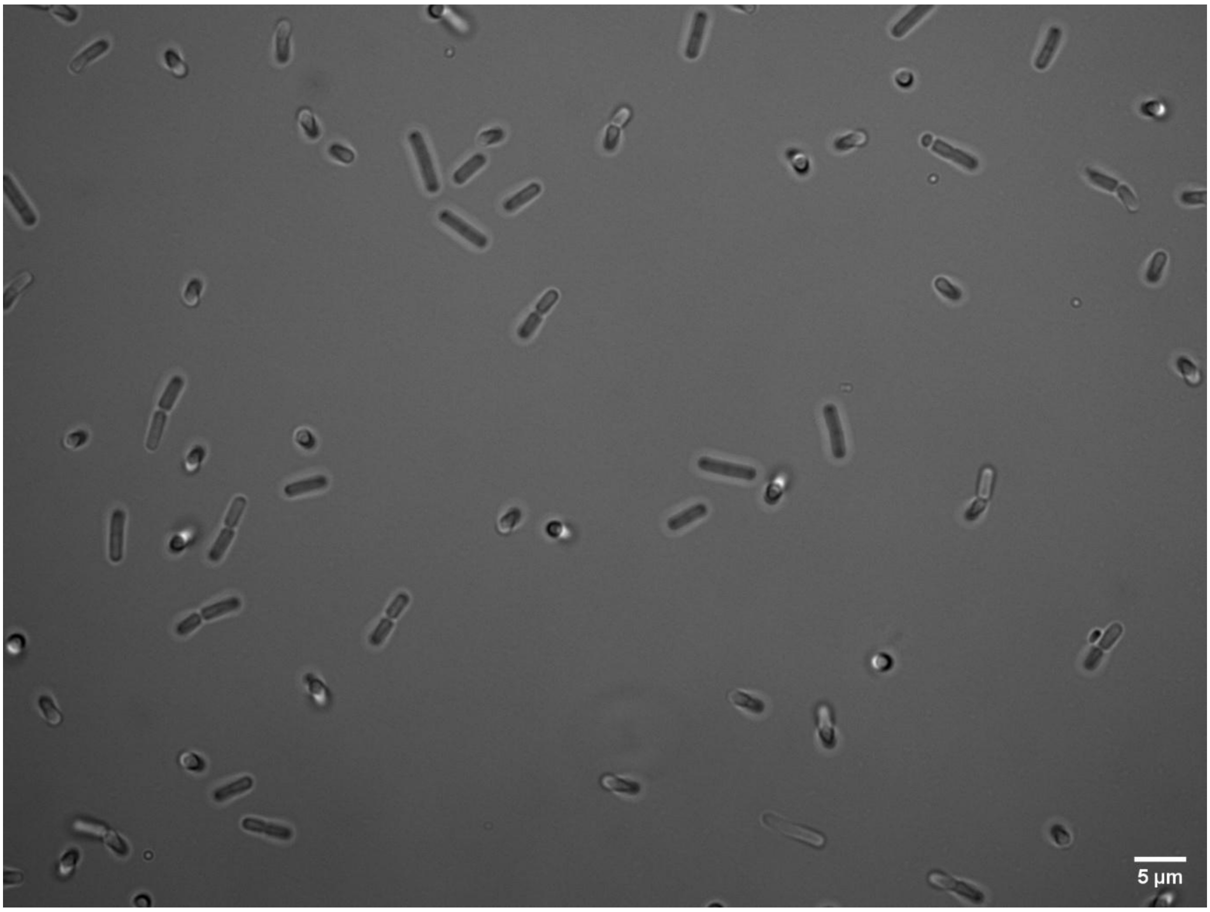

Figure S6. Spore germination and cell division of the *B. cereus* spores after disinfection with 5,000 ppm sodium hypochlorite (pH = 11.8). The field of view contains only vegetative and dividing bacterial cells and no spores, indicating the ineffectiveness of hypochlorite for disinfection of spores at this pH.

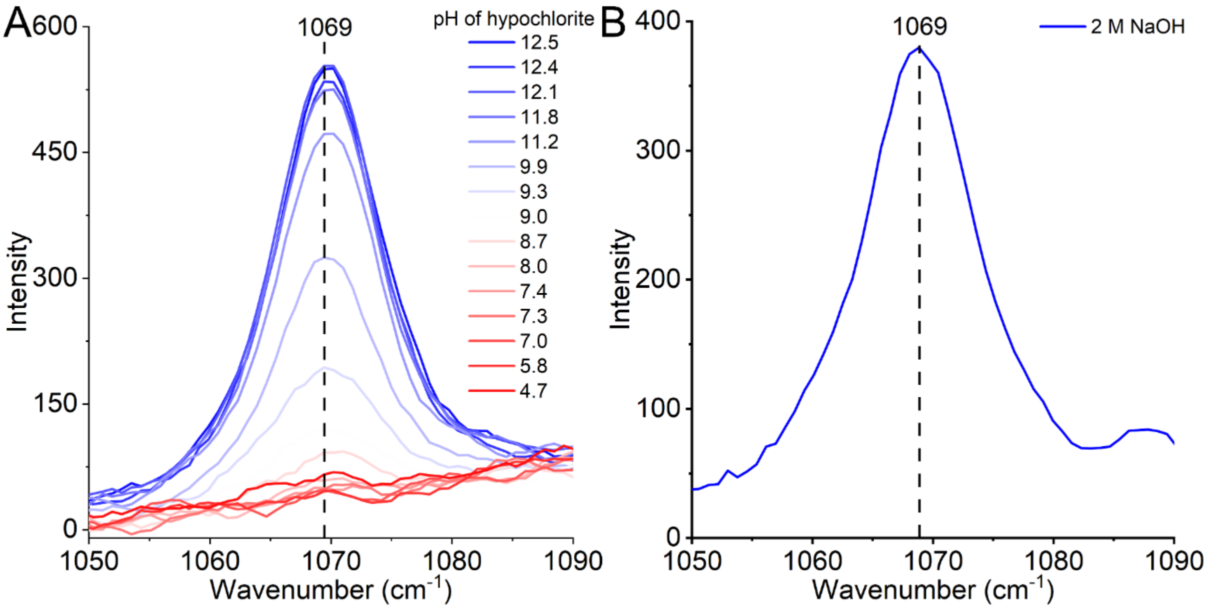

Figure S7. A) The evolution of the 1069 cm^{-1} Raman peak with pH. B) The Raman spectrum of 2 M aqueous NaOH.

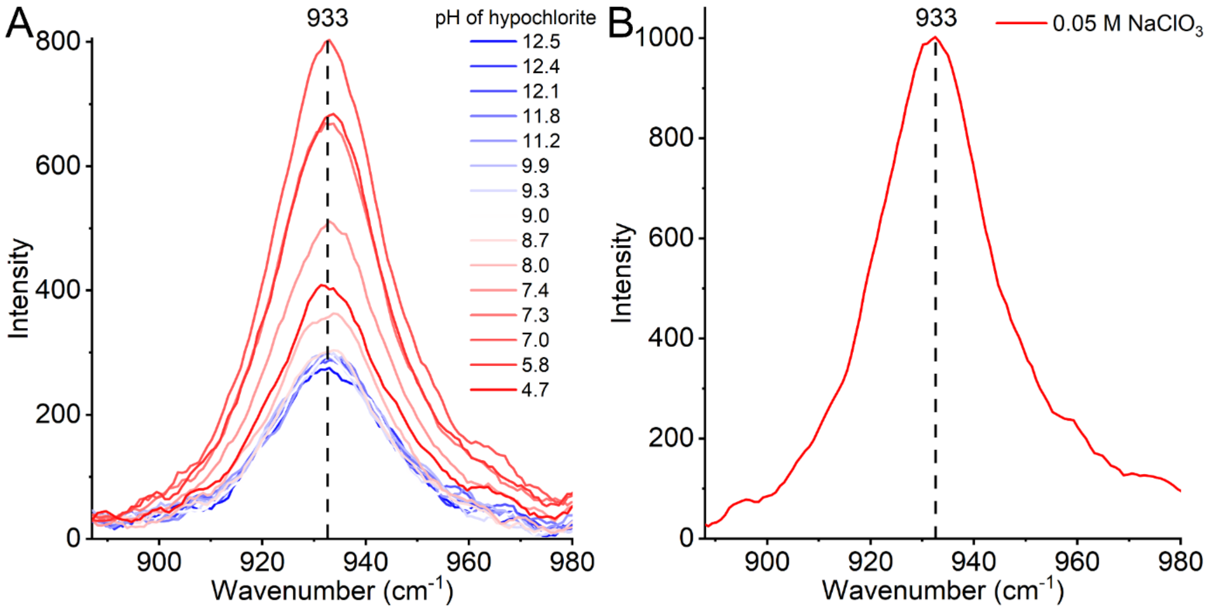

Figure S8. A) The evolution of the 933 cm^{-1} Raman peak with pH. B) The Raman spectrum of 0.05 M aqueous sodium chlorate.

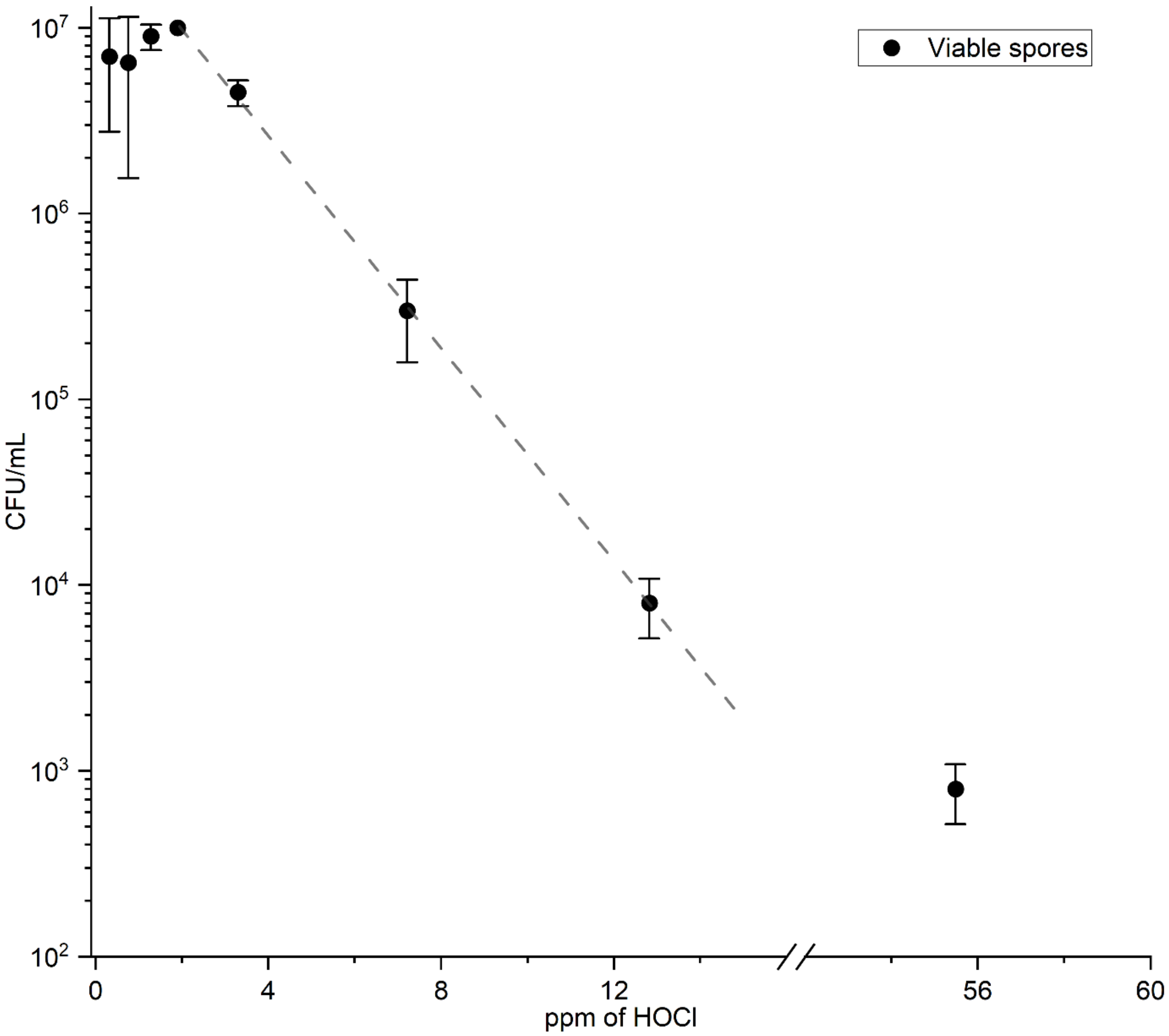

Figure S9. Viability of spores in relation to concentration of the HOCl form of hypochlorite. The same dataset from Figure 1 is presented here with pH values converted to relative concentration of HOCl in a 5,000 ppm hypochlorite solution.

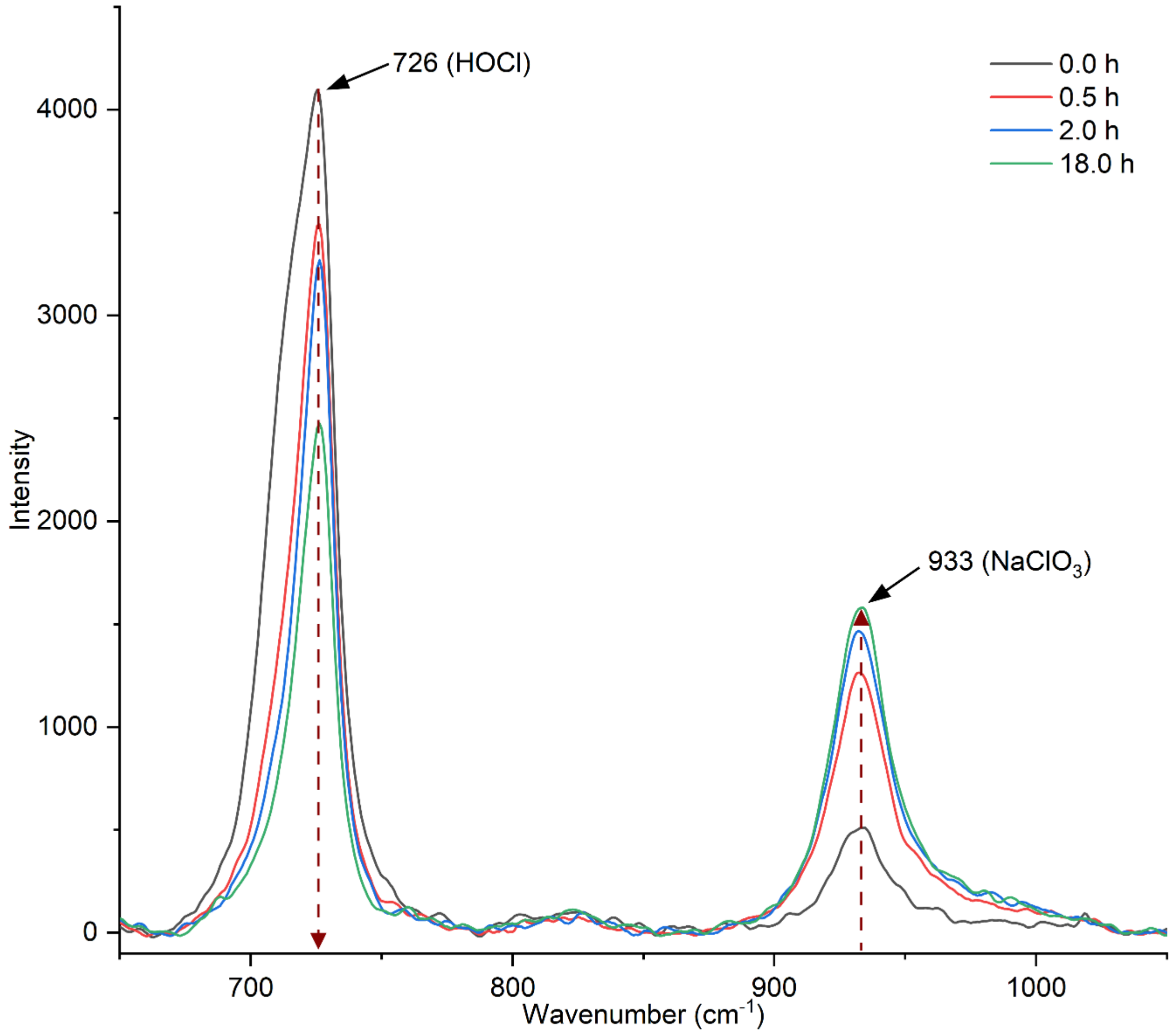

Figure S10. Raman spectra of sodium hypochlorite (pH 7.3) at different time intervals. A 50,000 ppm solution of sodium hypochlorite is prepared with a stating pH of 7.3 at 25°C and the Raman spectrum of this solution is measured at different time intervals. The spectra show increase in intensity of 933 cm^{-1} peak and concomitant decrease of 726 cm^{-1} peak. The changes in the intensities of these peaks implies that as a function of time HOCl dissociates to form NaOCl, indicating reduction in shelf life of hypochlorite solution at lower pH range.

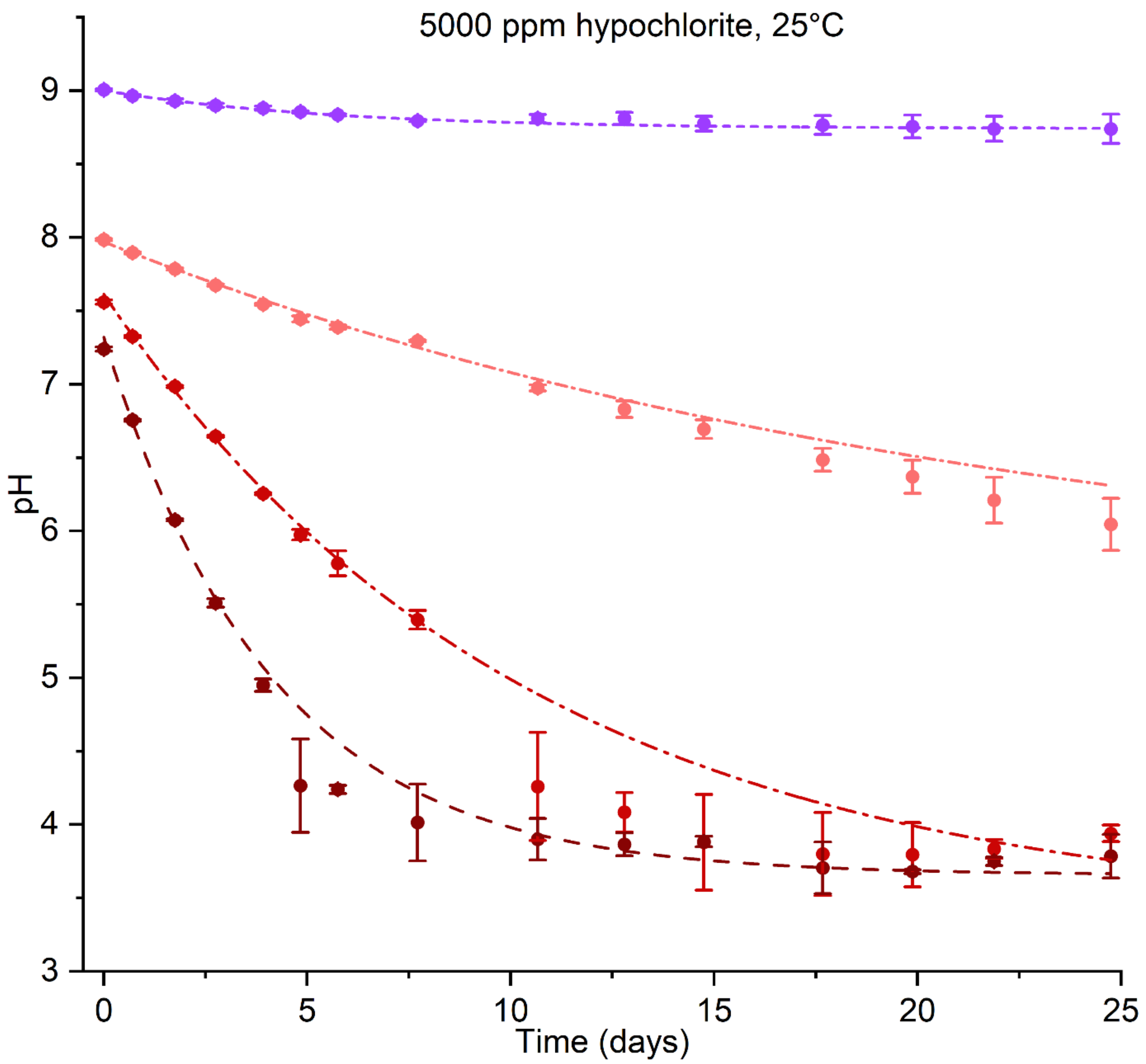

Figure S11. The change in pH of 5,000 ppm hypochlorite solution over time. The solutions are incubated at 25°C and pH is measured in regular intervals. The pH of the solution remains stable for initial pH of 9.0, however pH decreases over time for pH 8.0 and below. Comparison of the data with Figure 2D shows more than 100-fold difference in time scale of pH reduction compared to the stock (50,000 ppm) hypochlorite solution.

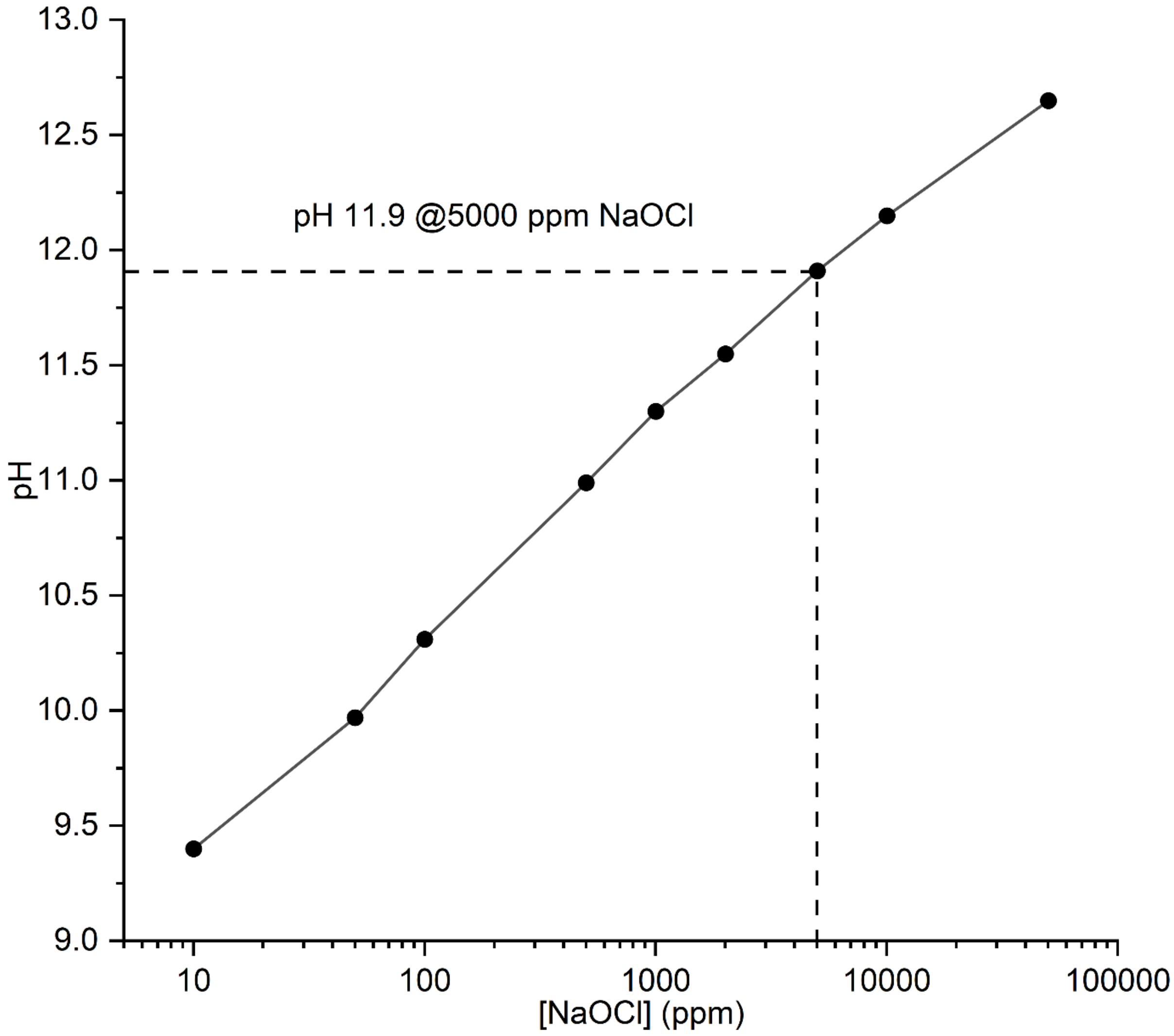

Figure S12. The pH of 5 % sodium hypochlorite as a function of dilution in water. The reduction in pH is indicative of conversion between NaOCl and HOCl. However, dilution to 5,000 ppm (used for our experiment) resulted in a pH of 11.9, indicating that hypochlorite existed exclusively in the NaOCl form. Diluting in water alone is insufficient for creating the HOCl necessary to disinfect spores.

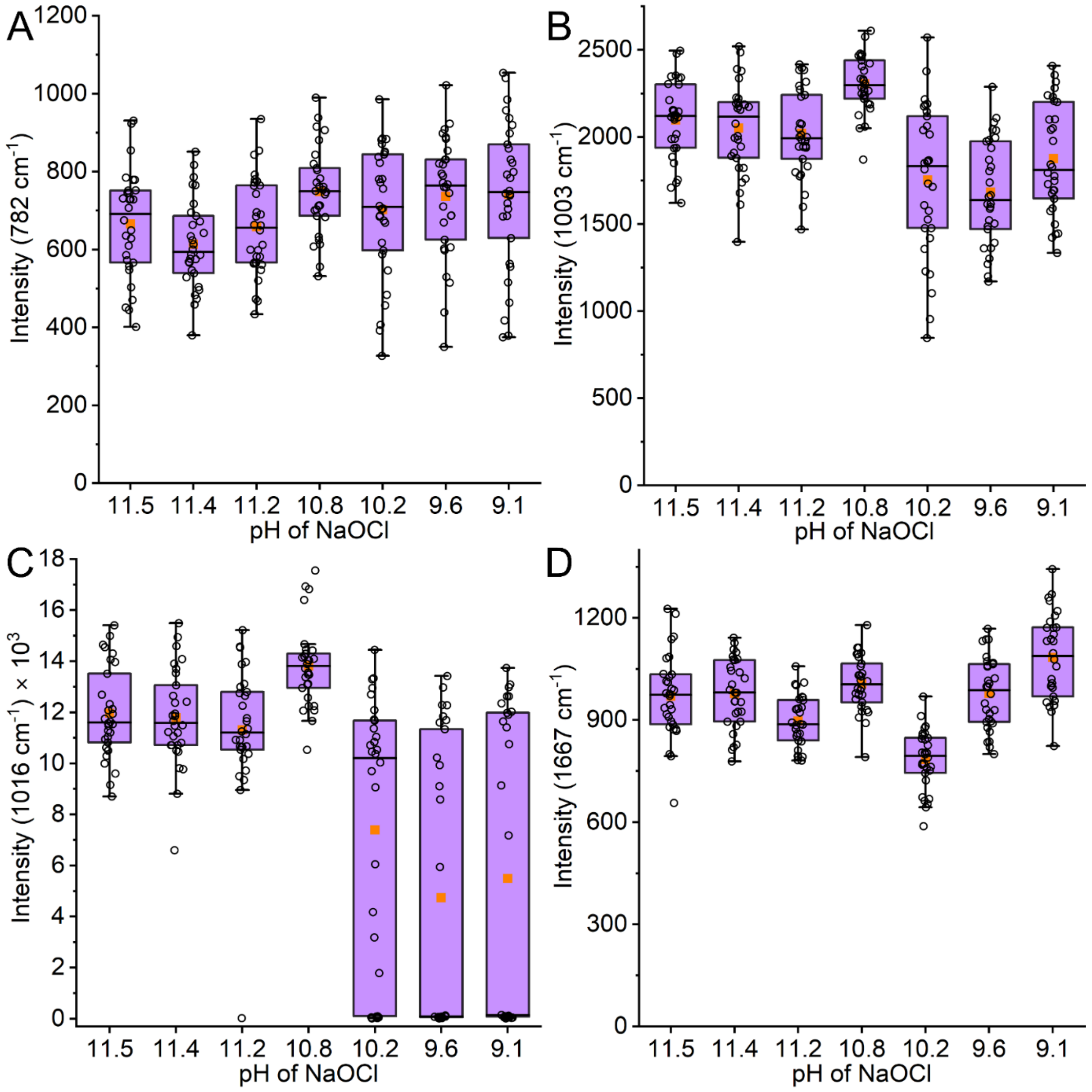

Figure S13. Intensity of characteristic Raman bands of spores after disinfection with hypochlorite at different pH levels. The measurements are performed by trapping individual spores in an optical trap, followed by Raman spectroscopy (n = 30). The band at 1016 cm^{-1} shows a bimodal distribution in Raman intensity after pH 10.8, with bimodality coefficients of 0.68, 0.77, and 0.78 respectively. This shows the complete loss of CaDPA from ~60 % of the spores. The orange dot is the average Raman intensity calculated from the measurement of 30 different spores.

Derivation of the relationship between pH and pK$_a$, and determination of pK$_a$ from Raman intensity of OCl$^-$ (713 cm^{-1}) and HOCl peaks (726 cm^{-1}).

The acid-base equilibrium of hypochlorous acid is given as:

$$HOCl \leftrightarrow OCl^- + H^+$$

The relationship between the pH of the solution, the acid dissociation constant (pK$_a$), and the ratio of the conjugate base (OCl^-) to the acid (HOCl) is given by the Henderson-Hasselbalch equation as:

$$pH = pk_a + \log\frac{[\mathrm{OCl^-}]}{[\mathrm{HOCl}]}$$

From this, the ratio can be expressed as:

$$\frac{[\mathrm{OCl^-}]}{[\mathrm{HOCl}]} = 10^{(p^H - p^{K_a})}$$

To express the OCl^- in terms of total concentration, the terms can be rearranged according to:

$$\frac{[\mathrm{OCl^-}\ ]}{(1 - [\mathrm{OCl^-}])} = 10^{(p^H - p^{K_a})}$$

This gives us the final expression to determine the pK$_a$ of hypochlorous acid by measuring the Raman intensity of the OCl^- and HOCl peaks.

$$[\mathrm{OCl^-}] = \frac{10^{(p^H - p^{K_a})}}{1+10^{(p^H - p^{K_a})}}.$$
